# Mr. Balfour's Speech at the Opening of Guy's Hospital Medical School

**Published:** 1913-06-07

**Authors:** 


### THE ENDOWMENT OF RESEARCH.

### Mr. Balfour's Speech at the Opening of Guy's Hospital Medical School.

The following is the speech delivered by Mr. A. J. Balfour at the opening of Guy's Hospital completed Medical and Dental Schools last Tuesday. The opening ceremony, over which Lord Goschen presided, was held in the physiological theatre. Mr. Balfour said:

Your Chairman has reminded you that fifteen years ago I had the pleasure and honour of speaking in this hall to the predecessors of the present students upon the claims which research had upon all who desire to see this country maintain its place among the nations in what is, after all, the greatest of the practical professions. In the fifteen years which have elapsed, this great block of buildings has been entirely reconstructed; £100,000 has been spent upon it, and you might perhaps be disposed to say that we can now look back on the work which has been done, congratulate ourselves upon its success, and rest from our labours. That is not the spirit in which I am going to address you. You have only just begun.

### Progressive Science Entails more Money.

The buildings, it is true, are finished; but a sum of money estimated at twice the amount which has been spent on the buildings is required before these buildings can do all they are expected to do in the interest of medicine, as medicine is now, and to make medicine what it will be in the lifetime of many whom I am addressing. This is a great task, but it is one not unworthy of the energy and liberality of our countrymen. Some unacquainted with the movement of modern science may ask why it is that in 1913 we require apparatus, buildings, and expenditure of all kinds infinitely in excess of what was necessary even fifty years ago. It is not merely the growth of a great urban population; it is the necessity that arises from the inherent progress of science itself. Look back fifty years, and you will find in the first place that some branches of science which were then, as now, studied by those who intended to devote their life to medicine, had, nevertheless, an incomparably smaller, or less obvious, connection with medicine than they have at the present time. Physics and biology and some parts of modern chemistry had not the close and intimate connection they now have. Physics and chemistry are intimately connected with all the physiological processes on which knowledge of life, of health, and of disease alike depend, and as the connection of other and collateral sciences is becoming closer with the science and practice of medicine, so the apparatus required has greatly increased in cost. The amount of knowledge required from teachers and the specialisation of teachers has itself grown until I sometimes wonder how it is possible for any physician in great practice even to keep abreast of what is being done in his own country and other countries which are engaged in a happy rivalry over the furtherance of knowledge—a rivalry far happier than the rivalry in armaments, but I sometimes think hardly less expensive.

### Functions of the Modern Hospital.

To the ordinary public the hospital seems simply to be a place where those who are sick or injured come for healing and relief. That is a very small and a very narrow view to take of its functions, because, unless the hospital was in addition to that a place where the future healers were to be trained, its functions must die out with the existing generation, and it would not provide for the carrying on of those great philanthropic duties for the benefit of future generations. It is at least as important that our great hospitals should be made admirable training grounds for the physicians and surgeons of the future as they should afford physicians and surgeons of the present a field for their beneficent enterprise. How is that to be accomplished? We must remember that to carry out this work effectively requires three separate kinds of action which are not necessarily associated in any single individual, and in many cases are not and cannot be. You have, in the first place, in the really successful professor of the healing art not only a knowledge of all that science of medicine can teach, but you must have that ready and apt intuition with power of diagnosis, with knowledge of human nature, which makes the physician a helper as well as a healer to those whom he has to attend.

### Where Progress May be Expected.

In the second place, you must have men who not only can do, but can explain. Then you want a man who can investigate. But what is not easy is to point out in what direction the next advance should be made, where progress may be expected, where Nature, under present and existing conditions, may be most easily compelled to yield up her secrets, and who may, by happy inspiration and genius, suggest a solution of some long-standing difficulty. That is the researcher. Genius is rare in any country and in any profession, and the medical profession cannot hope to have a larger share of it than others. All that organisation can do is to give to these rarely-endowed individuals some opportunity in which they can effectively exercise for the common advantage the gifts which God has given them. I am not sure that our present medical organisation does not need amendment in that respect. Men must live. If a man of research is going to devote to research hours which might profitably be given to general practice of his profession, you must have positions of security in which he can feel he is not sacrificing the interests of those nearest and dearest to him in the pursuit and advancement of new knowledge. Our system has grown up like most things in this country in a somewhat haphazard fashion. So rapid are the movements of modern thought, so absolutely necessary is it to differentiate more than we have done between the work of teaching and research, that I am certain the public must assist the great hospitals by a form of endowment which will enable them, when they get a man with a genius for research, to keep him and use him. (Cheers.)

What the Public Neither Knows nor Understands.

If there is to be a true organisation of medical research for the future you must find a place for this man. I make this appeal for liberality on the part of the general public. The actual suffering of the moment touches all our hearts. Take a man through the wards of a hospital and show him what is being done to diminish suffering and to restore a person to a happy, fruitful, and effective life and he is moved, and the most indifferent may open his purse-strings. The appeal I am making has no such natural advantages. I am appealing for the future, not for the present. I am appealing in favour of academic lines of research of which the public know, and understand, nothing, and yet upon which depends the real future of the healing art in this and other countries. The appeal is not easy to make; it is apt to fall on deaf and indifferent ears. But the future is as real as the present. Exercise your imagination a little and you will see it teaches you as much as a man who has broken his leg, and if only those who have the most will exercise that small effort of imagination I am quite certain that those who are responsible for this institution will find it as easy to collect the £200,000 which yet remains to be gathered in as they have been enabled, out of the liberality of the public and of the staff of the hospital, to obtain the money already collected for the buildings I now declare open.

